# Antibody limits *in vivo* murid herpesvirus-4 replication by IgG Fc receptor-dependent functions

**DOI:** 10.1099/vir.0.014266-0

**Published:** 2009-11

**Authors:** Debbie E. Wright, Susanna Colaco, Camilo Colaco, Philip G. Stevenson

**Affiliations:** 1 Division of Virology, Department of Pathology, University of Cambridge, UK; 2 Immunobiology Ltd, Babraham Research Campus, Cambridge, UK

## Abstract

Antibody is an important antiviral defence. However, it is considered to do little against human gamma-herpesviruses, which establish predominantly latent infections regulated by T cells. One limitation on analysing these infections has been that latency is already well-established at clinical presentation; early infection may still be accessible to antibody. Here, using murid herpesvirus-4 (MuHV-4), we tested the impact of adoptively transferred antibody on early gamma-herpesvirus infection. Immune sera and neutralizing and non-neutralizing monoclonal antibodies (mAbs) all reduced acute lytic MuHV-4 replication. The reductions, even by neutralizing mAbs, were largely or completely dependent on host IgG Fc receptors. Therefore, passive antibody can blunt acute gamma-herpesvirus lytic infection, and does this principally by IgG Fc-dependent functions rather than by neutralization.

## INTRODUCTION

Antibody provides an important defence against many epidemic viruses. Its protective efficacy correlates with the neutralization of cell-free virions (Zinkernagel & Hengartner, 2006). This is reflected epidemiologically in virus strains that differ mainly where neutralizing antibodies bind. Whether antibody is also effective against persistent pathogens such as gamma-herpesviruses is less clear. The archetypal gamma-herpesvirus, Epstein–Barr virus (EBV), causes disease mainly when latent; inhibiting its lytic replication during acute infectious mononucleosis makes little difference to latent viral loads (Yao *et al.*, 1989); also, long-term latent infection appears to be controlled by T cells (Rickinson & Moss, 1997). Therefore, antibody would seem to play little part in host defence. However, EBV infection takes at least 1 month to become symptomatic (Hoagland, 1964), by which time latency is already well-established. The early events in infection remain poorly defined. Substantial CD8^+^ T-cell responses to viral lytic antigens at clinical presentation (Callan *et al.*, 1996) suggest that significant lytic replication occurs early on, and vaccination with a viral lytic antigen can alleviate the symptoms of primary infection (Sokal *et al.*, 2007). Therefore, antibody might still be useful therapeutically in acute settings such as organ transplantation into EBV-naive recipients, where primary EBV infection confers a poor outcome (Walker *et al.*, 1995).

Genetic immunodeficiencies can provide clues as to what controls human viruses (Carneiro-Sampaio & Coutinho, 2007). However, B cell-deficient humans lack the main EBV latency reservoir and consequently fail to support normal persistence (Faulkner *et al.*, 1999). The contribution that antibody makes to host defence has therefore been hard to define. The failure of EBV to establish a significant experimental infection of non-primates further limits the opportunities for direct analysis. We and others (Virgin & Speck, 1999; Nash *et al.*, 2001; Stevenson & Efstathiou, 2005) have therefore developed murid herpesvirus-4 (MuHV-4) as a small-animal model of gamma-herpesvirus pathogenesis. MuHV-4 is genetically closer to Kaposi's sarcoma-associated herpesvirus than to EBV, but its tropism and pathogenesis are similar to those of EBV, and it currently provides the most tractable means to establish how antibody might interact with a gamma-herpesvirus *in vivo*.

T-cell depletion causes disease in B cell-deficient (Stewart *et al.*, 1998) but not immunocompetent (Stevenson *et al.*, 1999) MuHV-4 carriers, implying that antibody helps to control established infection. In support of this idea, adoptively transferred immune sera reduced MuHV-4 replication in B cell-deficient (Gangappa *et al.*, 2002) and T cell-depleted, CD28-deficient (Kim *et al.*, 2002) mice, and a rabbit serum raised against the MuHV-4 gp70 complement control protein reduced MuHV-4 replication in B cell-deficient mice (Gangappa *et al.*, 2002). However, human gamma-herpesviruses cause disease more by latency-associated tumours rather than by chronic lytic replication, so the main opportunity for antibody to protect against them is likely to be early on. The impact of antibody on early MuHV-4 replication has not been tested.

The mechanism of antibody-mediated protection was not addressed in the MuHV-4 studies listed above, but was speculated to be neutralization in one study (Kim *et al.*, 2002) and correlated with *in vitro* virus neutralization in the other (Gangappa *et al.*, 2002). However, gamma-herpesvirus neutralization is not well understood. For example, EBV can be neutralized *in vitro* (Thorley-Lawson & Geilinger, 1980), but EBV carriers continue to transmit infection with little evidence of viral antigenic variation, despite making virus-specific antibodies. Immune sera can block MuHV-4 infection of fibroblasts *in vitro* (Stevenson & Doherty, 1998), but they block *in vivo* host entry poorly (Gillet *et al.*, 2007a) and tend to enhance MuHV-4 infection of IgG Fc receptor-positive (FcR^+^) cells such as macrophages (Rosa *et al.*, 2007). Thus, whilst immune sera can block virions binding to fibroblasts (Gill *et al.*, 2006), virion membrane fusion seems to remain intact. Poor *in vivo* neutralization could consequently reflect the availability of additional binding routes that are harder for antibody to block. mAbs to gH–gL and gB can block infection post-binding and achieve a more universal neutralization (Gill *et al.*, 2006; Gillet *et al.*, 2006), but gH–gL and gB are poorly immunogenic, so this activity of immune sera is weak (Gillet *et al.*, 2007b). The virion fusion machinery – gH and gB – is also protected by glycosylation (Gillet & Stevenson, 2007a) and by gB and gH–gL associating on the virion surface (Gillet & Stevenson, 2007a, b; Gillet *et al.*, 2009). The gB–gH–gL complex appears to unfold only after a post-endocytic dissociation of gL (Gillet *et al.*, 2008a, b), so extracellular gB and gH can remain protected. Another consideration is that *in vivo* gamma-herpesvirus lytic spread may involve direct cell–cell contact more than cell-free virion release, and thereby resist neutralization as described for other herpesviruses (Hooks *et al.*, 1976).

We asked here whether immune sera or monoclonal antibodies (mAbs) could reduce the primary lytic replication of MuHV-4 in immunocompetent hosts and, if so, how they might act. Our aim was to define a basis for antibody-based interventions in primary gamma-herpesvirus infections.

## METHODS

### Mice.

BALB/c, C57BL/6, 129Sv (Harlan UK) and 129Sv×B6 FcR*γ*^−/−^Fc*γ*RII^−/−^ mice (Taconic Europe) were bred in the Cambridge University Department of Pathology animal unit and infected with MuHV-4 when 6–12 weeks old. Intranasal infections were performed in a volume of 30 μl, given under general anaesthesia. For luciferase imaging, mice were injected intraperitoneally with luciferin (2 mg per mouse), anaesthetized with isoflurane, then scanned with an IVIS Lumina (Caliper Life Sciences). Quantitative comparisons used the total radiance (photons s^−1^ cm^−2^) over each region of interest, relative to a negative-control region. All experiments conformed to local animal-ethics regulations and to Home Office Project Licence 80/1992.

### Sera and mAbs.

Immune sera were collected at least 3 months post-infection and pooled from 10–12 mice each. Hybridomas were derived from MuHV-4-immune mice by fusion with NS0 myeloma cells (Köhler & Milstein, 1975), except for mAb DW-6F6, which was derived from mice infected with influenza A/PR/8/34 and is specific for the influenza H1 haemagglutinin. Antibodies were concentrated from hybridoma supernatants by ammonium sulfate precipitation, dialysed against PBS, isotyped by ELISA (Sigma) and quantified by Mancini assay (Mancini *et al.*, 1965). Those used for *in vivo* protection are listed in Table 1.

### Cells and viruses.

BHK-21 fibroblasts (ATCC CCL-10) and NMuMG epithelial cells (CRL-1636) were grown in Dulbecco's modified Eagle's medium (DMEM) with 2 mM glutamine, 100 U penicillin ml^−1^, 100 μg streptomycin ml^−1^ (all from Sigma) and 5 % fetal calf serum (PAA Laboratories). Hybridomas were grown in RPMI medium (Sigma), supplemented as for DMEM. All viruses were derived from a MuHV-4 bacterial artificial chromosome-cloned genome (Adler *et al.*, 2000). The wild type and its luciferase-expressing derivative (Milho *et al.*, 2009) were grown and titrated in BHK-21 cells (de Lima *et al.*, 2004). Infected cells were freeze–thawed and virions concentrated by high-speed centrifugation (38 000 ***g***, 90 min). Cell debris was then removed by low-speed centrifugation (1000 ***g***, 10 min).

### Virus titres and neutralization assays.

MuHV-4 was titrated by plaque assay on BHK-21 cells (de Lima *et al.*, 2004). Infectious virus in *ex vivo* tissues was measured by plaque assay of freeze–thawed tissue homogenates. To determine virus titre in noses, we removed a block of tissue bounded by the cartilaginous tip of the nose anteriorly, the orbits posteriorly, the zygomatic arches laterally, the palate ventrally and the nasal bones dorsally. Cell monolayers were incubated with virus (2 h, 37 °C), overlaid with 0.3 % carboxymethylcellulose and, 4 days later, fixed (4 % formaldehyde) and stained (0.1 % toluidine blue) for plaque counting. Titres were compared statistically between experimental groups by unpaired, two-tailed *t*-tests, assuming equal variance. The calculations were performed in Excel. *P* values of <0.05 were considered statistically significant.

### Flow cytometry.

MuHV-4-infected cells (2 p.f.u. per cell, 18 h) were trypsinized, washed in PBS and incubated (1 h, 4 °C) with MuHV-4 glycoprotein-specific mAbs, followed by fluorescein-conjugated rabbit anti-mouse IgG pAb (Dako Cytomation). The cells were washed twice in PBS after each incubation and analysed on a FACSCalibur (BD Biosciences).

### Immunoblotting.

*N*-Linked glycans were removed with protein *N*-glycanase F; *O*-linked glycans were removed with sialidase A+*O*-glycanase according to the manufacturer's instructions (Prozyme). Deglycosylated and intact samples were denatured (95 °C, 5 min) in Laemmli's buffer [60 mM Tris-Cl (pH 6.8); 3 % (v/v) SDS; 5 % (v/v) 2-mercaptoethanol; 10 % (v/v) glycerol; 0.002 % (v/v) bromophenol blue], resolved by SDS-PAGE and transferred to PVDF membranes. The membranes were blocked with 10 % non-fat milk and probed with MuHV-4-specific mAbs plus horseradish peroxidase-conjugated rabbit anti-mouse IgG pAb (Dako Cytomation). Membranes were washed four times in PBS/0.1 % Tween 20 after each step. Bound antibody was visualized by ECL substrate development (APBiotech).

## RESULTS

### Immune sera reduce MuHV-4 replication in naive hosts

**Fig. 1. f1:**
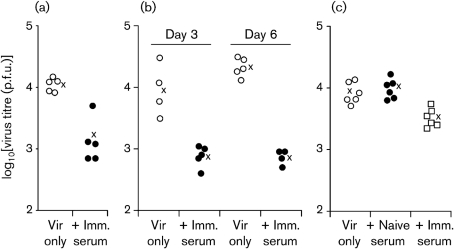
Protection against MuHV-4 replication by immune sera. (a) C57BL/6 mice were given 3×10^4^ p.f.u. MuHV-4 intranasally (i.n.) and at the same time treated or not with 200 μl immune serum intraperitoneally (i.p.). Infectious virus in lungs was measured by plaque assay 3 days later. Each point shows the titre for one mouse; × shows mean values. Immune serum reduced lung titres significantly (*P*<0.0005 by two-tailed Student's *t*-test). (b) C57BL/6 mice were infected and treated or not with immune serum as in (a). The effects of immune serum on day 3 and day 6 titres were then compared. The reduction in titre was significant at day 6 (*P*<0.0001), but not at day 3 (*P*=0.06). (c) BALB/c mice were infected and given immune serum as in (a), or naive serum as an additional control. Virus titres in lungs were determined after 5 days. Immune serum reduced titres significantly compared with either virus only or naive serum (*P*<0.01). Naive serum had no effect compared with virus only (*P*=0.5).

Adoptively transferred immune sera can reduce MuHV-4 replication in persistently infected mice that lack normal antibody production (Gangappa *et al.*, 2002; Kim *et al.*, 2002). We wanted to know whether immune sera could also reduce acute viral replication in naive, immunocompetent hosts. We tested this by giving MuHV-4-immune sera intraperitoneally to naive mice at the same time as intranasal MuHV-4 virions (Fig. 1). Significant reductions in virus titres were observed in both C57BL/6 (Fig. 1 a, b[Fig f1]) and BALB/c (Fig. 1c[Fig f1]) mice and across multiple time points. Naive sera had no effect (Fig. 1c[Fig f1]).

**Fig. 2. f2:**
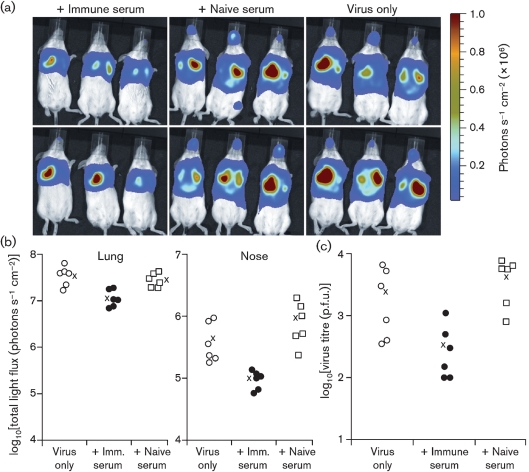
Immune serum limits MuHV-4 lytic gene expression in lungs and noses. (a) BALB/c mice were infected i.n. with luciferase^+^ MuHV-4 (3×10^4^ p.f.u.) and at the same time given immune serum (200 μl), naive serum (200 μl) or nothing i.p. Virus replication was monitored by luciferin injection and CCD camera scanning at 5 days post-infection. (b) Quantification of signals shown in (a). Each point shows the luciferase signal of one mouse; × shows mean values. Immune serum reduced luciferase expression significantly compared with naive serum (*P*<0.02 for noses, *P*<0.004 for lungs) or no-serum controls (*P*<0.03 for noses, *P*<0.006 for lungs) using Student's two-tailed *t*-test. (c) Noses from (a) were titrated for infectious virus by plaque assay. Each point shows the titre for one mouse. Immune serum reduced titres significantly compared with naive serum (*P*<0.005). Titres with immune serum were also lower than with virus only, although the wide scatter of values made this difference non-significant by *t*-test (*P*=0.06).

We used luciferase-positive MuHV-4 (Milho *et al.*, 2009) as an alternative means of tracking *in vivo* lytic infection (Fig. 2a, b[Fig f2]). Immune sera again reduced significantly the extent of acute host colonization. This was evident both in lungs and in noses. Plaque assays (Fig. 2c[Fig f2]) confirmed a reduction in nose infection by immune serum. In other experiments, we obtained similar data for noses and lungs, but nose titres were generally more variable between individual mice.

### Non-neutralizing mAbs reduce MuHV-4 replication in naive hosts

**Fig. 3. f3:**
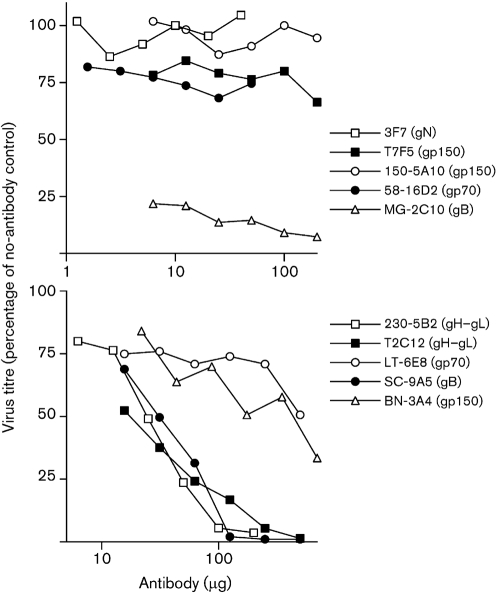
*In vitro* neutralization of MuHV-4 virions by mAbs. MuHV-4 virions (100 p.f.u.) were incubated with mAbs and then plaque-assayed on BHK-21 cells. The highest amounts of each antibody used for neutralization correspond to the amounts given per mouse. Small changes in infectivity (<3-fold reduction) were considered not significant because of the potential for effects such as virion cross-linking to operate *in vitro*. Thus, only mAbs MG-2C10, T2C12, 230-5B2 and SC-9A5 were considered to be neutralizing. Consistent results were obtained in at least two experiments for each mAb.

**Fig. 4. f4:**
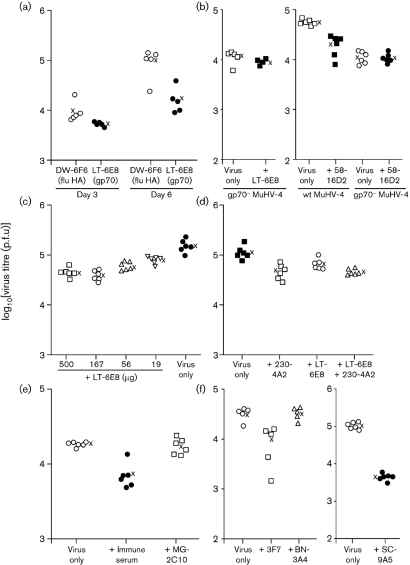
Reduction in *in vivo* MuHV-4 infectivity by glycoprotein-specific mAbs. (a) C57BL/6 mice were infected i.n. with MuHV-4 (3×10^4^ p.f.u.) and at the same time given antibody i.p. (500 μg). Infectious virus titres in lungs were then determined by plaque assay. Each point shows the titre for one mouse; × shows mean values. The gp70-specific mAb LT-6E8 reduced virus titres significantly at day 5 (*P*<0.005 by Student's two-tailed *t*-test) compared with the influenza haemagglutinin (flu HA)-specific control, although not at day 3 (*P*=0.1). (b) In an equivalent experiment, mAb LT-6E8 did not affect the replication of gp70^−^ MuHV-4 (*P*=0.2). mAb 58-16D2 (50 μg), which recognizes a different domain of gp70, also significantly reduced the 5 day post-infection titres of wild-type (wt; *P*<0.00002) but not gp70^−^ (*P*=0.8) MuHV-4. (c) BALB/c mice were infected i.n. with MuHV-4 (3×10^4^ p.f.u.) and at the same time given mAb LT-6E8 i.p. in differing amounts. Infectious virus titres in lungs were determined by plaque assay 5 days later. Each point shows the titre for one mouse; × shows mean values. All LT-6E8 amounts reduced virus titres compared with the no-antibody control (*P*<0.003); 500 μg was marginally more effective than 56 or 19 μg (*P*<0.04), but no more effective than 167 μg (*P*=0.3). (d) BALB/c mice were infected i.n. with MuHV-4 (3×10^4^ p.f.u.) and at the same time given mAb LT-6E8 (500 μg) or mAb 230-4A2 (500 μg) i.p., both together or no antibody. Infectious virus titres in lungs were determined by plaque assay 5 days later. All mAb treatments reduced virus titres significantly compared with the no-antibody control (*P*<0.02). Both LT-6E8 and 230-4A2 together were more effective than LT-6E8 alone (*P*<0.03), but not more effective than 230-4A2 alone (*P*=0.8). (e) C57BL/6 mice were infected i.n. as in (a), and the effect of immune serum (200 μl) was compared with that of the neutralizing gB-specific IgM mAb MG-2C10 (200 μg). Immune serum reduced virus titres significantly (*P*<0.0001), whereas MG-2C10 had no effect (*P*=0.7). (f) Mice were infected i.n. as in (a) and at the same time given antibody or not i.p. (results of two separate experiments are shown). Infectious virus in lungs was titrated by plaque assay 5 days later. mAbs 3F7 (IgG_2a_, anti-gN, non-neutralizing, 40 μg) and SC-9A5 (IgG_3_, anti-gB, neutralizing, 500 μg) reduced virus titres (*P*<0.002), whereas mAb BN-3A4 (IgG_1_, anti-gp150, non-neutralizing, 700 μg) did not (*P*=0.9).

Since the complexity of immune sera makes mechanisms of protection difficult to establish, we used mAbs to explore further the interaction between antibody and MuHV-4. Table 1 summarizes the results. Fig. 3 shows neutralization assays. In Fig. 4(a), mAb LT-6E8 reduced MuHV-4 replication compared with a control mAb (influenza haemagglutinin-specific). LT-6E8 recognizes gp70, the main product of MuHV-4 ORF4 (Gillet *et al.*, 2007c) and the target used by Gangappa *et al.* (2002) for protection by immune serum. mAb 58-16D2, which recognizes a different gp70 domain (Gillet *et al.*, 2007c), was also effective (Fig. 4b[Fig f4]). As expected, neither LT-6E8 nor 58-16D2 affected the replication of MuHV-4 lacking ORF4 (Fig. 4b[Fig f4]).

The amounts of mAb used depended on what each hybridoma produced. In general, we used the maximum amount possible, so as not to miss a therapeutic effect. We also ensured that at least 200 μg of any ineffective mAb was given, so as to be sure that ineffectiveness did not reflect low antibody concentration. Titration of LT-6E8 (Fig. 4c[Fig f4]), a standard effective mAb, showed that even 20 μg antibody reduced MuHV-4 replication significantly.

LT-6E8 blocks heparan sulfate binding by gp70, but does not neutralize (Fig. 3), because virions can also bind to heparan sulfate via gH–gL. mAb 230-4A2 blocks heparan sulfate binding by gH–gL and so neutralizes synergistically with LT-6E8 (Gillet *et al.*, 2008c). However, LT-6E8 and 230-4A2 gave no synergistic reduction in *in vivo* virus titres (Fig. 4d[Fig f4]). mAb MG-2C10 (Gillet *et al.*, 2006), a neutralizing, gB-specific IgM (Fig. 3), had no effect on *in vivo* virus replication (Fig. 4e[Fig f4]). mAbs 3F7 (gN-specific, non-neutralizing) (May *et al.*, 2005a) and SC-9A5 (gB-specific, neutralizing) both reduced *in vivo* lytic replication, whilst mAb BN-3A4 (gp150-specific, non-neutralizing) did not (Fig. 4f[Fig f4]). Therefore, no correlation, either positive or negative, was evident between an antibody's capacity to neutralize cell-free virions *in vitro* and its capacity to reduce virus replication *in vivo*.

### Reduced lytic infection by antibody depends on host IgG Fc receptors

**Fig. 5. f5:**
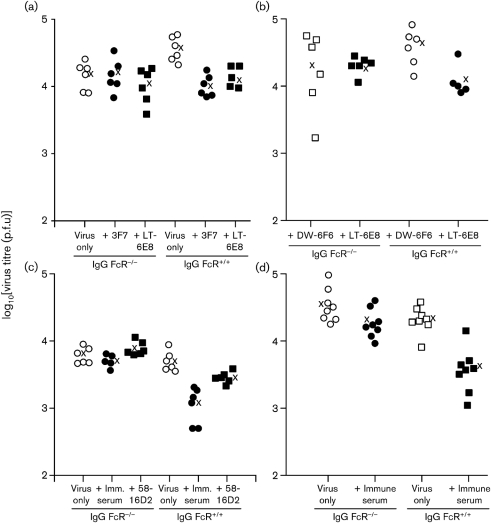
Antibody-mediated protection by non-neutralizing mAbs and by immune serum is IgG Fc receptor-dependent. (a) FcR*γ*^−/−^Fc*γ*RII^−/−^ mice (IgG FcR^−/−^) were compared with 129Sv (IgG FcR^+/+^) controls for antibody-dependent protection against MuHV-4. Mice were infected i.n. (3×10^4^ p.f.u.) and at the same time given antibody or not i.p. Infectious virus in lungs was titrated 5 days later by plaque assay. Each point shows the titre for one mouse; × shows mean values. mAbs 3F7 (40 μg) and LT-6E8 (500 μg) both reduced virus titres significantly in FcR^+/+^ (*P*<0.02 by Student's two-tailed *t*-test) but not FcR^−/−^ (*P*=0.3) mice. (b) IgG FcR^−/−^ or C57BL/6 (IgG FcR^+/+^) mice were infected i.n. as in (a) and at the same time given i.p. 500 μg LT-6E8 or DW-6F6 (anti-influenza haemagglutinin). Lungs were titrated for infectious virus 5 days later. mAb LT-6E8 reduced titres significantly in IgG FcR^+/+^ (*P*<0.05) but not IgG FcR^−/−^ (*P*=0.5) mice compared with the control. (c) IgG FcR^−/−^ and 129Sv IgG FcR^+/+^ mice were compared by lung virus titre 5 days after i.n. MuHV-4 infection and i.p. injection of either nothing (virus only), immune serum (200 μl) or mAb 58-16D2 (50 μg). Immune serum reduced virus titres significantly in IgG FcR^+/+^ (*P*<0.001) but not IgG FcR^−/−^ (*P*=0.3) mice. mAb 58-16D2 also reduced virus titres significantly in IgG FcR^+/+^ (*P*<0.02) but not IgG FcR^−/−^ mice. (d) IgG FcR^−/−^ and C57BL/6 IgG FcR^+/+^ mice were compared by lung virus titre 5 days after i.n. MuHV-4 infection and i.p. injection of either nothing (virus only) or immune serum (200 μl). Immune serum again reduced virus titres significantly in IgG FcR^+/+^ (*P*<0.0002) but not IgG FcR^−/−^ (*P*=0.08) mice.

The antiviral effect of IgG occurs principally by Fab-dependent virion neutralization and by Fc-dependent recruitment of other immune effectors. Recruiting cellular effectors requires signalling through the common IgG Fc receptor gamma chain (FcR*γ*) (Nimmerjahn & Ravetch, 2008). We therefore explored the mechanism of antibody-mediated protection by infecting FcR*γ*^−/−^ mice. We used mice that also lacked the FcgRII inhibitory receptor (FcR*γ*^−/−^Fc*γ*RII^−/−^), so as to reduce any excess immunosuppressive effect of IgG binding to cells lacking activating Fc receptors. mAbs 3F7 and LT-6E8 both failed to reduce virus titres significantly in the FcR^−^ mice, whereas they were effective in FcR^+^ 129Sv controls (Fig. 5a[Fig f5]). We obtained an equivalent result (Fig. 5b[Fig f5]) with C57BL/6 controls (the FcR^−^ mice were 129Sv×C57BL/6) with mAb 58-16D2 (Fig. 5c[Fig f5]), and with immune sera using both 129Sv and C57BL/6 controls (Fig. 5c, d[Fig f5]). Therefore protection by both non-neutralizing mAbs and immune sera depended on IgG Fc receptors.

### Neutralization has at most a minor role in antibody-mediated MuHV-4 control

**Fig. 6. f6:**
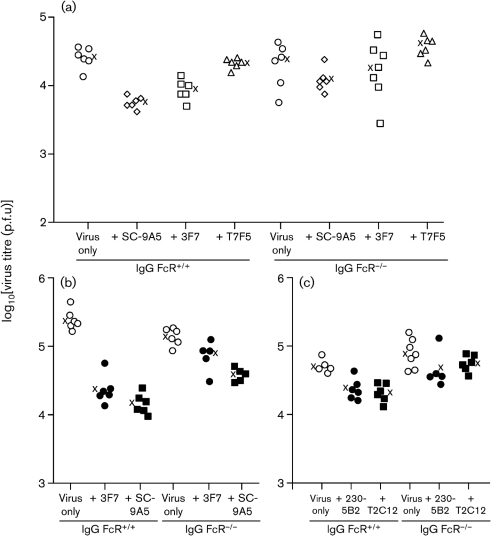
Antibody-mediated protection by neutralizing mAbs is also largely IgG Fc receptor-dependent. (a) C57BL/6 (IgG FcR^+/+^) or FcR*γ*^−/−^Fc*γ*RII^−/−^ (IgG FcR^−/−^) mice were infected with MuHV-4 i.n. (3×10^4^ p.f.u.) and given antibody or not i.p. Five days later, infectious virus in lungs was titrated by plaque assay. Each point shows the titre for one mouse; × shows mean values. IgG FcR^+/+^ mice showed a significant reduction in virus replication by mAbs SC-9A5 (500 μg) and 3F7 (40 μg) (*P*<0.001 by Student's two-tailed *t*-test), but not by mAb T7F5 (200 μg) (*P*=0.2). None of the mAbs reduced virus replication significantly in IgG FcR^−/−^ mice. (b) A repeat experiment again showed a significant reduction in IgG FcR^+/+^ lung titres by mAbs 3F7 (40 μg) and SC-9A5 (500 μg) (*P*<0.0003). This time the reduction in IgG FcR^−/−^ lung titres by SC-9A5, whilst lower than that of IgG FcR^+/+^ (3.7-fold rather than 17-fold), was also significant (*P*<0.0003), as was that by 3F7 (*P*<0.02). (c) IgG FcR^+/+^ and IgG FcR^−/−^ mice were further compared for virus titre reductions by the neutralizing gH–gL-specific mAbs T2C12 (500 μg) and 230-5B2 (200 μg), using the same infection protocol as in (a). Both mAbs reduced lung virus titres significantly in IgG FcR^+/+^ (*P*<0.005) but not IgG FcR^−/−^ (*P*>0.13) mice.

As mAb SC-9A5 is an IgG_3_, it should not engage strongly with IgG Fc receptors (Nimmerjahn & Ravetch, 2008). Therefore, as a neutralizing mAb (Fig. 3), it might be expected to reduce virus titres equally in FcR^−^ and FcR^+^ mice. In one experiment (Fig. 6a[Fig f6]), SC-9A5 reduced the virus titres of FcR^−^ mice, but not to a significant level; in a repeat experiment (Fig. 6b[Fig f6]), the reduction was significant, although there was also a significant reduction by mAb 3F7 (*P*<0.02), something not seen in other experiments (e.g. Figs 5a[Fig f5], 6a[Fig f6]). In both experiments, SC-9A5 gave a substantially greater reduction in FcR^+^ than FcR^−^ mice (4.6-fold rather than 1.9-fold in Fig. 6a[Fig f6]; 17-fold rather than 3.7-fold in Fig. 6b[Fig f6]).

We examined further a possible contribution of neutralization to *in vivo* titre reductions with the gH–gL-specific neutralizing mAbs T2C12 and 230-5B2 (Fig. 6c[Fig f6]). Again, there were substantial reductions in FcR^+^ mouse titres, and only small reductions that did not reach statistical significance in FcR^−^ mouse titres. Therefore, even neutralizing mAbs acted *in vivo* largely through IgG Fc receptor engagement.

### gp150 is not an effective target for MuHV-4 control by mAbs

**Fig. 7. f7:**
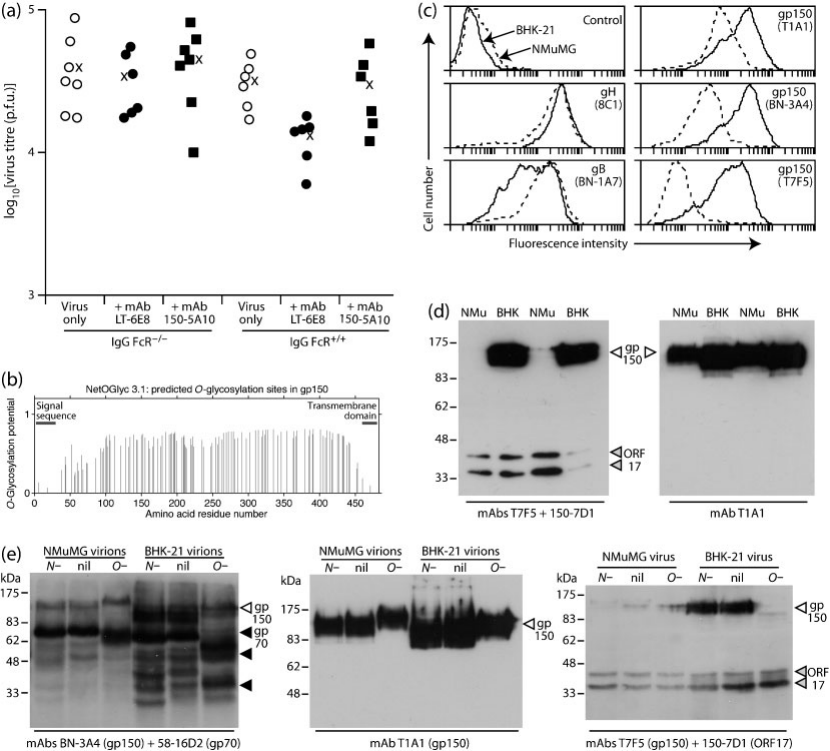
*O*-Glycosylation may make gp150 a poor therapeutic antibody target. (a) C57BL/6 (IgG FcR^+/+^) or FcR*γ*^−/−^Fc*γ*RII^−/−^ (IgG FcR^−/−^) mice were infected i.n. with MuHV-4 (3×10^4^ p.f.u.) and given antibody or not i.p. Five days later, infectious virus in lungs was titrated by plaque assay. Each point shows the titre for one mouse; × shows mean values. IgG FcR^+/+^ mice showed a significant reduction in virus replication by mAb LT-6E8 (500 μg) (*P*<0.002), but not by mAb 150-5A10 (200 μg) (*P*=0.9), using Student's two-tailed *t*-test. Neither mAb reduced virus replication significantly in IgG FcR^−/−^ mice. (b) Predicted *O*-glycosylation sites in gp150 (Julenius *et al.*, 2005). The predicted signal sequence (residues 1–22) and transmembrane domain (residues 459–481) are also shown. (c) Flow-cytometric analysis of BHK-21 cells (solid lines) and NMuMG cells (dashed lines) infected with MuHV-4 (2 p.f.u. per cell, 18 h). Control=secondary antibody only. mAbs 8C1 (anti-gH) and BN-1A7 (anti-gB) provide controls for the level of infection. None of the mAbs stained uninfected cells. Staining with mAb 150-5A10 was equivalent to that of BN-3A4. (d) Immunoblot of virions derived from NMuMG cells (NMu) or BHK-21 cells (BHK). Each blot shows two independently grown virus stocks from each cell type. mAb 150-7D1 recognizes the ORF17 capsid component and provides a loading control. (e) Virions derived from NMuMG cells or BHK-21 cells were untreated (nil), digested with PNGase F to remove *N*-linked glycans (*N*−) or digested with sialidase+*O*-glycanase to remove *O*-linked glycans (*O*−). Samples were then immunoblotted for gp150. mAb 150-7D1 (ORF17) provides a loading control; mAb 58-16D2 demonstrates *O*-linked glycan removal from gp70 in the same samples.

gp150 is expressed abundantly on MuHV-4-infected fibroblasts and epithelial cells. Therefore, it was surprising that the gp150-specific mAbs BN-3A4 (Fig. 4f[Fig f4]), T7F5 (Fig. 6a[Fig f6]) and 150-5A10 (Fig. 7a[Fig f7]) all failed to reduce virus titres. These mAbs all recognize epitopes contained within gp150 aa 108–151 (Gillet *et al.*, 2007b). mAb T1A1, which recognizes an epitope in the region 151–270, also gave no reduction (data not shown).

gp150 is predicted to be heavily *O*-glycosylated (Fig. 7b[Fig f7]). All of the gp150-specific mAbs used recognize glycan-independent epitopes, as they recognize gp150 fragments expressed in *Escherichia coli* (Gillet *et al.*, 2007b and data not shown), but glycans could potentially hide their epitopes. We have found previously (Gillet & Stevenson, 2007a) that *O*-glycosylation inhibits gB recognition by mAb MG-2C10. This manifests as virions from NMuMG epithelial cells resisting recognition by MG-2C10 unless *O*-glycans are removed, whereas virions from BHK-21 fibroblasts are recognized with or without deglycosylation. (Cells of epithelial origin presumably add more extensive, mucin-like *O*-glycans.) Flow cytometry of MuHV-4-infected NMuMG cells showed less staining than infected BHK-21 cells for all gp150-specific mAbs (Fig. 7c[Fig f7]). There was also less gp150 detected by immunoblot of NMuMG cell-derived compared with BHK-21 cell-derived virions (Fig. 7d[Fig f7]). The mAbs used recognize no products of the corresponding gp150^−^ or gp70^−^ viruses; the bands below 70 kDa in Fig. 7(e) are all alternative glycoforms of gp70 (Gillet *et al.*, 2007c). Reduced gp150 detection on NMuMG cell-derived virions could reflect a lower gp150 content. However, these virions do not show the typical gp150-deficient phenotype (de Lima *et al.*, 2004) of heparan sulfate-independent infection (data not shown). Moreover, gp150 recognition was not reduced uniformly: recognition by T1A1 was relatively preserved, whereas recognition by T7F5 was reduced markedly. Therefore, it seemed more likely that gp150 recognition was being masked.

gp150 has at least some some *O*-linked glycans, because it is immunoprecipitated readily by jacalin (May *et al.*, 2005b). Removing *O*-linked glycans did not increase convincingly the detection of NMuMG-cell-derived gp150 (Fig. 7e[Fig f7]). However, the detection of BHK-21 cell-derived gp150 decreased markedly, presumably because gp150 became unstable (or was otherwise lost) when its *O*-linked glycans were removed. (Note that the actual molecular mass of gp150 is very difficult to infer from SDS-PAGE: *O*-glycan digestion retards its migration even further.) The greater equivalence of NMuMG and BHK-21 gp150 signals after *O*-linked glycan removal suggested that glycosylation substantially modified recognition by gp150-specific mAbs. Therefore, differential glycosylation could explain the inefficacy of gp150-specific mAbs in reducing *in vivo* virus titres.

## DISCUSSION

Passive antibody, given promptly, attenuates primary alpha-herpesvirus infections (Whitley, 1994; Ogilvie, 1998). Gamma-herpesviruses are generally viewed as less accessible to antibody-based interventions, as they colonize their hosts largely by latency-associated lymphoproliferation. We show here that immune sera and mAbs can nonetheless reduce MuHV-4 acute lytic replication and that they act predominantly by IgG Fc receptor engagement.

Antibody can protect mice against herpes simplex virus (HSV) both by neutralization and by IgG Fc receptor-dependent functions (McKendall, 1985; Ishizaka *et al.*, 1995). Early studies mainly compared neutralizing and non-neutralizing mAbs. Whilst antibodies capable of neutralization need not act by neutralization, the finding that glycoprotein C was a viable target for antibody *in vivo* and not for neutralization (Balachandran *et al.*, 1982; Kümel *et al.*, 1985) argued that, here at least, protection involved host Fc receptors. Protection by immune serum was subsequently shown to be IgG Fc receptor-dependent (Chu *et al.*, 2008). The difficulty with interpreting such results is that the HSV gE–gI binds human but not mouse IgG Fc (Johansson *et al.*, 1985). In contrast, MuHV-4 naturally infects mice (Kozuch *et al.*, 1993) and gamma-herpesviruses are not known to express IgG Fc receptors. Our findings with MuHV-4 – i.e. that non-neutralization epitopes provided effective antibody targets *in vivo* and that protection by immune serum was largely IgG Fc receptor-dependent – therefore suggested that antibodies generally act via Fc receptors in host defence against herpesviruses.

One difference between MuHV-4 and HSV is that the HSV gD provides a good neutralization target (Minson *et al.*, 1986); the analogous (although non-homologous) MuHV-4 gp150 does not, and seems to function instead in antibody evasion. gp150 is abundant on infected-cell surfaces, but gp150-specific mAbs failed to reduce *in vivo* virus replication. Some uncertainty remains over gp150 processing: the equivalent gene of most other gamma-herpesviruses can be spliced (Beisel *et al.*, 1985) to exclude some of the region equivalent to the immunogenic ‘stalk’ of gp150 (Gillet *et al.*, 2007b). However, we have found no evidence of gp150 splicing by RT-PCR, either *in vitro* or *in vivo* (data not shown). A mAb recognizing the C-terminal part of the gp150 extracellular domain identifies additional, faster-migrating forms on SDS-PAGE, suggesting the possibility of post-translational cleavage (data not shown), but we have not detected corresponding N-terminal fragments in infected-cell supernatants, so these may simply be different glycoforms. Also, gp150 cleavage alone would not explain gp150-specific mAbs being ineffective, as gp70 is shed abundantly into infected-cell supernatants (Kapadia *et al.*, 1999; Gillet *et al.*, 2007c) yet remained a good antibody target. Differential gp150 glycosylation seems more likely to limit the impact of gp150-specific mAbs.

The importance of IgG Fc receptors in limiting *in vivo* MuHV-4 replication was consistent with MuHV-4 neutralization being much more difficult than *in vitro* assays of fibroblast infection would suggest (Gillet *et al.*, 2007a). One consideration is virions blocked for fibroblast infection by immune sera using IgG Fc receptors to productively infect myeloid cells (Smith *et al.*, 2007). However, there was no evidence for neutralization being more effective in FcR*γ*^−/−^ than in FcR*γ*^+/+^ mice. Therefore, a more important consideration is probably the *in vivo* accessibility of virions to antibody. gp48, which promotes direct MuHV-4 cell–cell spread via actin polymerization (Gill *et al.*, 2008), contributes more to *in vivo* virus replication (May *et al.*, 2005c) than does gp150, which functions in virion release (de Lima *et al.*, 2004), or gL, which functions in virion cell binding (Gillet *et al.*, 2007d). Similarly, HSV cell–cell spread via gE contributes significantly to dissemination *in vivo* but not *in vitro* (Dingwell *et al.*, 1994), whilst cell binding by gp50 is important for pseudorabies virus dissemination *in vitro*, yet is largely dispensable *in vivo* (Peeters *et al.*, 1993). Thus, herpesviruses seem generally to remain cell-associated *in vivo*, affording limited opportunities for neutralization. As the main function of cell-free virions is genome transmission between hosts, their release need not extend beyond apical epithelial cells. The difficulty of herpesvirus neutralization may limit what passive antibody can achieve – certainly the inhibition of MuHV-4 replication achieved here was relatively modest. Further study is required to determine whether this reflects a specific mechanism of viral evasion.

Which IgG Fc receptor-dependent effector mechanism limits MuHV-4 replication remains unclear. Antibody-dependent cytotoxicity seems the most likely. Depleting natural killer (NK) cells with mAb PK136 (anti-NK1.1) had no effect on antibody-mediated protection (data not shown). However, macrophages, monocytes or granulocytes may be more important mediators of such cytotoxicity in mice (Nimmerjahn & Ravetch, 2008). Another (non-exclusive) possibility is enhanced virion uptake by dendritic cells improving T-cell priming. Thus, the failure of gp150-specific antibodies to reduce viral titres could have reflected that their opsonization allows particularly efficient dendritic-cell infection after uptake, and therefore disruption of antigen presentation (Smith *et al.*, 2007; Gillet *et al.*, 2007b), as opposed to virion uptake without infection, which should allow efficient antigen presentation. The *in vivo* fate of opsonized virions and infected cells clearly requires further study. The present data indicate that it is this, rather than neutralization, that chiefly determines the therapeutic effect of antiviral antibodies.

Might passive antibody also be effective against EBV? Whilst unlikely to prevent infection, it could potentially be helpful acutely in immunosuppressed patients exposed to EBV by reducing the extent of lytic viral spread. Antibody might also be a useful adjunct to latency-directed therapies such as adoptive T-cell transfer. Most considerations of anti-EBV antibody have focused on gp350 because anti-gp350 mAbs can block B-cell infection *in vitro* (Thorley-Lawson & Geilinger, 1980). The ineffectiveness of mAbs against the MuHV-4 gp150 suggested that other targets might be better *in vivo*.

## Figures and Tables

**Table 1. t1:** Summary of mAbs used *in vivo*

**Antibody**	**Target**	**Isotype**	Neutralization*	***In vivo* titre†**
LT-6E8	gp70 (SCR1+2)	IgG_2b_	−	+
58-16D2	gp70 (SCR4)	IgG_2a_	−	+
3F7	gN	IgG_2a_	−	+
SC-9A5	gB	IgG_3_	++	++
MG-2C10	gB	IgM	+	−
BN-3A4	gp150	IgG_1_	−	−
150-5A10	gp150	IgG_2a_	−	−
T7F5	gp150	IgG_2a_	−	−
T1A1	gp150	IgG_2a_	−	−
230-5B2	gH–gL	IgG_2a_	+	+
T2C12	gH–gL	IgG_2a_	+	+
230-4A2	gH–gL	IgG_2a_	+	+

*Plaque reduction on BHK-21 cells, scored from negative (−) to strong (++). †Reduction in lung titre by plaque assay, scored from negative (−) to strong (++).

## References

[r1] Adler, H., Messerle, M., Wagner, M. & Koszinowski, U. H. (2000). Cloning and mutagenesis of the murine gammaherpesvirus 68 genome as an infectious bacterial artificial chromosome. J Virol 74, 6964–6974. 10888635 10.1128/jvi.74.15.6964-6974.2000 PMC112213

[r2] Balachandran, N., Bacchetti, S. & Rawls, W. E. (1982). Protection against lethal challenge of BALB/c mice by passive transfer of monoclonal antibodies to five glycoproteins of herpes simplex virus type 2. Infect Immun 37, 1132–1137. 6290390 10.1128/iai.37.3.1132-1137.1982 PMC347658

[r3] Beisel, C., Tanner, J., Matsuo, T., Thorley-Lawson, D., Kezdy, F. & Kieff, E. (1985). Two major outer envelope glycoproteins of Epstein–Barr virus are encoded by the same gene. J Virol 54, 665–674. 2987520 10.1128/jvi.54.3.665-674.1985 PMC254850

[r4] Callan, M. F., Steven, N., Krausa, P., Wilson, J. D., Moss, P. A., Gillespie, G. M., Bell, J. I., Rickinson, A. B. & McMichael, A. J. (1996). Large clonal expansions of CD8^+^ T cells in acute infectious mononucleosis. Nat Med 2, 906–911. 8705861 10.1038/nm0896-906

[r5] Carneiro-Sampaio, M. & Coutinho, A. (2007). Immunity to microbes: lessons from primary immunodeficiencies. Infect Immun 75, 1545–1555. 17283094 10.1128/IAI.00787-06 PMC1865715

[r6] Chu, C. F., Meador, M. G., Young, C. G., Strasser, J. E., Bourne, N. & Milligan, G. N. (2008). Antibody-mediated protection against genital herpes simplex virus type 2 disease in mice by Fc gamma receptor-dependent and -independent mechanisms. J Reprod Immunol 78, 58–67. 17950908 10.1016/j.jri.2007.08.004 PMC2441821

[r7] de Lima, B. D., May, J. S. & Stevenson, P. G. (2004). Murine gammaherpesvirus 68 lacking gp150 shows defective virion release but establishes normal latency *in vivo*. J Virol 78, 5103–5112. 15113892 10.1128/JVI.78.10.5103-5112.2004 PMC400354

[r8] Dingwell, K. S., Brunetti, C. R., Hendricks, R. L., Tang, Q., Tang, M., Rainbow, A. J. & Johnson, D. C. (1994). Herpes simplex virus glycoproteins E and I facilitate cell-to-cell spread *in vivo* and across junctions of cultured cells. J Virol 68, 834–845. 8289387 10.1128/jvi.68.2.834-845.1994 PMC236520

[r9] Faulkner, G. C., Burrows, S. R., Khanna, R., Moss, D. J., Bird, A. G. & Crawford, D. H. (1999). X-linked agammaglobulinemia patients are not infected with Epstein–Barr virus: implications for the biology of the virus. J Virol 73, 1555–1564. 9882361 10.1128/jvi.73.2.1555-1564.1999 PMC103980

[r10] Gangappa, S., Kapadia, S. B., Speck, S. H. & Virgin, H. W. (2002). Antibody to a lytic cycle viral protein decreases gammaherpesvirus latency in B-cell-deficient mice. J Virol 76, 11460–11468. 12388707 10.1128/JVI.76.22.11460-11468.2002 PMC136779

[r11] Gill, M. B., Gillet, L., Colaco, S., May, J. S., de Lima, B. D. & Stevenson, P. G. (2006). Murine gammaherpesvirus-68 glycoprotein H–glycoprotein L complex is a major target for neutralizing monoclonal antibodies. J Gen Virol 87, 1465–1475. 16690911 10.1099/vir.0.81760-0

[r12] Gill, M. B., Edgar, R., May, J. S. & Stevenson, P. G. (2008). A gamma-herpesvirus glycoprotein complex manipulates actin to promote viral spread. PLoS ONE 3, e1808 18350146 10.1371/journal.pone.0001808 PMC2262946

[r13] Gillet, L. & Stevenson, P. G. (2007a). Antibody evasion by the N terminus of murid herpesvirus-4 glycoprotein B. EMBO J 26, 5131–5142. 18034158 10.1038/sj.emboj.7601925 PMC2094095

[r14] Gillet, L. & Stevenson, P. G. (2007b). Evidence for a multi-protein gamma-2-herpesvirus entry complex. J Virol 81, 13082–13091. 17898071 10.1128/JVI.01141-07 PMC2169126

[r15] Gillet, L., Gill, M. B., Colaco, S., Smith, C. M. & Stevenson, P. G. (2006). Murine gammaherpesvirus-68 glycoprotein B presents a difficult neutralization target to monoclonal antibodies derived from infected mice. J Gen Virol 87, 3515–3527. 17098966 10.1099/vir.0.82313-0 PMC2884974

[r16] Gillet, L., May, J. S. & Stevenson, P. G. (2007a). Post-exposure vaccination improves gammaherpesvirus neutralization. PLoS One 2, e899 17878934 10.1371/journal.pone.0000899 PMC1964807

[r17] Gillet, L., May, J. S., Colaco, S. & Stevenson, P. G. (2007b). The murine gammaherpesvirus-68 gp150 acts as an immunogenic decoy to limit virion neutralization. PLoS One 2, e705 17684552 10.1371/journal.pone.0000705 PMC1931612

[r18] Gillet, L., Adler, H. & Stevenson, P. G. (2007c). Glycosaminoglycan interactions in murine gammaherpesvirus-68 infection. PLoS One 2, e347 17406671 10.1371/journal.pone.0000347 PMC1829177

[r19] Gillet, L., May, J. S., Colaco, S. & Stevenson, P. G. (2007d). Glycoprotein L disruption reveals two functional forms of the murine gammaherpesvirus 68 glycoprotein H. J Virol 81, 280–291. 17050601 10.1128/JVI.01616-06 PMC1797276

[r20] Gillet, L., Colaco, S. & Stevenson, P. G. (2008a). The murid herpesvirus-4 gL regulates an entry-associated conformation change in gH. PLoS One 3, e2811 18665235 10.1371/journal.pone.0002811 PMC2481400

[r21] Gillet, L., Colaco, S. & Stevenson, P. G. (2008b). Glycoprotein B switches conformation during murid herpesvirus 4 entry. J Gen Virol 89, 1352–1363. 18474550 10.1099/vir.0.83519-0 PMC2886948

[r22] Gillet, L., Colaco, S. & Stevenson, P. G. (2008c). The murid herpesvirus-4 gH/gL binds to glycosaminoglycans. PLoS One 3, e1669 18301747 10.1371/journal.pone.0001669 PMC2253500

[r23] Gillet, L., Alenquer, M., Glauser, D. L., Colaco, S., May, J. S. & Stevenson, P. G. (2009). Glycoprotein L sets the neutralization profile of murid herpesvirus-4. J Gen Virol 90, 1202–1214. 19264603 10.1099/vir.0.008755-0 PMC2885041

[r24] Hoagland, R. J. (1964). The incubation period of infectious mononucleosis. Am J Public Health Nations Health 54, 1699–1705. 14240492 10.2105/ajph.54.10.1699 PMC1255045

[r25] Hooks, J. J., Burns, W., Hayashi, K., Geis, S. & Notkins, A. L. (1976). Viral spread in the presence of neutralizing antibody: mechanisms of persistence in foamy virus infection. Infect Immun 14, 1172–1178. 185150 10.1128/iai.14.5.1172-1178.1976 PMC415510

[r26] Ishizaka, S. T., Piacente, P., Silva, J. & Mishkin, E. M. (1995). IgG subtype is correlated with efficiency of passive protection and effector function of anti-herpes simplex virus glycoprotein D monoclonal antibodies. J Infect Dis 172, 1108–1111. 7561190 10.1093/infdis/172.4.1108

[r27] Johansson, P. J. H., Myhre, E. B. & Blomberg, J. (1985). Specificity of Fc receptors induced by herpes simplex virus type 1: comparison of immunoglobulin G from different animal species. J Virol 56, 489–494. 2997471 10.1128/jvi.56.2.489-494.1985 PMC252604

[r28] Julenius, K., Mølgaard, A., Gupta, R. & Brunak, S. (2005). Prediction, conservation analysis and structural characterization of mammalian mucin-type *O*-glycosylation sites. Glycobiology 15, 153–164. 15385431 10.1093/glycob/cwh151

[r29] Kapadia, S. B., Molina, H., van Berkel, V., Speck, S. H. & Virgin, H. W. (1999). Murine gammaherpesvirus 68 encodes a functional regulator of complement activation. J Virol 73, 7658–7670. 10438856 10.1128/jvi.73.9.7658-7670.1999 PMC104293

[r30] Kim, I. J., Flaño, E., Woodland, D. L. & Blackman, M. A. (2002). Antibody-mediated control of persistent gamma-herpesvirus infection. J Immunol 168, 3958–3964. 11937552 10.4049/jimmunol.168.8.3958

[r31] Köhler, G. & Milstein, C. (1975). Continuous cultures of fused cells secreting antibody of predefined specificity. Nature 256, 495–497. 1172191 10.1038/256495a0

[r32] Kozuch, O., Reichel, M., Lesso, J., Remenová, A., Labuda, M., Lysy, J. & Mistríková, J. (1993). Further isolation of murine herpesviruses from small mammals in southwestern Slovakia. Acta Virol 37, 101–105. 8105644

[r33] Kümel, G., Kaerner, H. C., Levine, M., Schröder, C. H. & Glorioso, J. C. (1985). Passive immune protection by herpes simplex virus-specific monoclonal antibodies and monoclonal antibody-resistant mutants altered in pathogenicity. J Virol 56, 930–937. 2415719 10.1128/jvi.56.3.930-937.1985 PMC252666

[r34] Mancini, G., Carbonara, A. O. & Heremans, J. F. (1965). Immunochemical quantitation of antigens by single radial immunodiffusion. Immunochemistry 2, 235–254. 4956917 10.1016/0019-2791(65)90004-2

[r35] May, J. S., Colaco, S. & Stevenson, P. G. (2005a). Glycoprotein M is an essential lytic replication protein of the murine gammaherpesvirus 68. J Virol 79, 3459–3467. 15731240 10.1128/JVI.79.6.3459-3467.2005 PMC1075704

[r36] May, J. S., Coleman, H. M., Boname, J. M. & Stevenson, P. G. (2005b). Murine gammaherpesvirus-68 ORF28 encodes a non-essential virion glycoprotein. J Gen Virol 86, 919–928. 15784886 10.1099/vir.0.80661-0

[r37] May, J. S., Walker, J., Colaco, S. & Stevenson, P. G. (2005c). The murine gammaherpesvirus 68 ORF27 gene product contributes to intercellular viral spread. J Virol 79, 5059–5068. 15795291 10.1128/JVI.79.8.5059-5068.2005 PMC1069585

[r38] McKendall, R. R. (1985). IgG-mediated viral clearance in experimental infection with herpes simplex virus type 1: role for neutralization and Fc-dependent functions but not C′ cytolysis and C5 chemotaxis. J Infect Dis 151, 464–470. 2982965 10.1093/infdis/151.3.464

[r39] Milho, R., Smith, C. M., Marques, S., Alenquer, M., May, J. S., Gillet, L., Gaspar, M., Efstathiou, S., Simas, J. P. & Stevenson, P. G. (2009). *In vivo* imaging of murid herpesvirus-4 infection. J Gen Virol 90, 21–32. 19088269 10.1099/vir.0.006569-0 PMC2885022

[r40] Minson, A. C., Hodgman, T. C., Digard, P., Hancock, D. C., Bell, S. E. & Buckmaster, E. A. (1986). An analysis of the biological properties of monoclonal antibodies against glycoprotein D of herpes simplex virus and identification of amino acid substitutions that confer resistance to neutralization. J Gen Virol 67, 1001–1013. 2423636 10.1099/0022-1317-67-6-1001

[r41] Nash, A. A., Dutia, B. M., Stewart, J. P. & Davison, A. J. (2001). Natural history of murine gamma-herpesvirus infection. Philos Trans R Soc Lond B Biol Sci 356, 569–579. 11313012 10.1098/rstb.2000.0779 PMC1088445

[r42] Nimmerjahn, F. & Ravetch, J. V. (2008). Fc*γ* receptors as regulators of immune responses. Nat Rev Immunol 8, 34–47. 18064051 10.1038/nri2206

[r43] Ogilvie, M. M. (1998). Antiviral prophylaxis and treatment in chickenpox. A review prepared for the UK Advisory Group on Chickenpox on behalf of the British Society for the Study of Infection. J Infect 36 (Suppl. 1), 31–38. 9514106 10.1016/s0163-4453(98)80153-9

[r44] Peeters, B., Pol, J., Gielkens, A. & Moormann, R. (1993). Envelope glycoprotein gp50 of pseudorabies virus is essential for virus entry but is not required for viral spread in mice. J Virol 67, 170–177. 8380069 10.1128/jvi.67.1.170-177.1993 PMC237349

[r45] Rickinson, A. B. & Moss, D. J. (1997). Human cytotoxic T lymphocyte responses to Epstein–Barr virus infection. Annu Rev Immunol 15, 405–431. 9143694 10.1146/annurev.immunol.15.1.405

[r46] Rosa, G. T., Gillet, L., Smith, C. M., de Lima, B. D. & Stevenson, P. G. (2007). IgG Fc receptors provide an alternative infection route for murine gamma-herpesvirus-68. PLoS One 2, e560 17593961 10.1371/journal.pone.0000560 PMC1891442

[r47] Smith, C. M., Gill, M. B., May, J. S. & Stevenson, P. G. (2007). Murine gammaherpesvirus-68 inhibits antigen presentation by dendritic cells. PLoS One 2, e1048 17940612 10.1371/journal.pone.0001048 PMC2002512

[r48] Sokal, E. M., Hoppenbrouwers, K., Vandermeulen, C., Moutschen, M., Léonard, P., Moreels, A., Haumont, M., Bollen, A., Smets, F. & Denis, M. (2007). Recombinant gp350 vaccine for infectious mononucleosis: a phase 2, randomized, double-blind, placebo-controlled trial to evaluate the safety, immunogenicity, and efficacy of an Epstein–Barr virus vaccine in healthy young adults. J Infect Dis 196, 1749–1753. 18190254 10.1086/523813

[r49] Stevenson, P. G. & Doherty, P. C. (1998). Kinetic analysis of the specific host response to a murine gammaherpesvirus. J Virol 72, 943–949. 9444986 10.1128/jvi.72.2.943-949.1998 PMC124564

[r50] Stevenson, P. G. & Efstathiou, S. (2005). Immune mechanisms in murine gammaherpesvirus-68 infection. Viral Immunol 18, 445–456. 16212523 10.1089/vim.2005.18.445

[r51] Stevenson, P. G., Cardin, R. D., Christensen, J. P. & Doherty, P. C. (1999). Immunological control of a murine gammaherpesvirus independent of CD8^+^ T cells. J Gen Virol 80, 477–483. 10073710 10.1099/0022-1317-80-2-477

[r52] Stewart, J. P., Usherwood, E. J., Ross, A., Dyson, H. & Nash, T. (1998). Lung epithelial cells are a major site of murine gammaherpesvirus persistence. J Exp Med 187, 1941–1951. 9625754 10.1084/jem.187.12.1941 PMC2212355

[r53] Thorley-Lawson, D. A. & Geilinger, K. (1980). Monoclonal antibodies against the major glycoprotein (gp350/220) of Epstein–Barr virus neutralize infectivity. Proc Natl Acad Sci U S A 77, 5307–5311. 6254073 10.1073/pnas.77.9.5307 PMC350047

[r54] Virgin, H. W. & Speck, S. H. (1999). Unraveling immunity to *γ*-herpesviruses: a new model for understanding the role of immunity in chronic virus infection. Curr Opin Immunol 11, 371–379. 10448140 10.1016/s0952-7915(99)80063-6

[r55] Walker, R. C., Paya, C. V., Marshall, W. F., Strickler, J. G., Wiesner, R. H., Velosa, J. A., Habermann, T. M., Daly, R. C. & McGregor, C. G. (1995). Pretransplantation seronegative Epstein–Barr virus status is the primary risk factor for posttransplantation lymphoproliferative disorder in adult heart, lung, and other solid organ transplantations. J Heart Lung Transplant 14, 214–221. 7779838

[r56] Whitley, R. J. (1994). Neonatal herpes simplex virus infections: is there a role for immunoglobulin in disease prevention and therapy? Pediatr Infect Dis J 13, 432–438. 8072834

[r57] Yao, Q. Y., Ogan, P., Rowe, M., Wood, M. & Rickinson, A. B. (1989). The Epstein–Barr virus : host balance in acute infectious mononucleosis patients receiving acyclovir anti-viral therapy. Int J Cancer 43, 61–66. 2536008 10.1002/ijc.2910430114

[r58] Zinkernagel, R. M. & Hengartner, H. (2006). Protective ‘immunity’ by pre-existent neutralizing antibody titers and preactivated T cells but not by so-called ‘immunological memory’. Immunol Rev 211, 310–319. 16824138 10.1111/j.0105-2896.2006.00402.x

